# Fatal monomorphic ventricular tachycardia in a semi-urban setting in Cameroon: a case report

**DOI:** 10.1186/s13104-017-2501-4

**Published:** 2017-05-08

**Authors:** Clovis Nkoke, Engelbert Bain Luchuo, Laetitia Dikoume

**Affiliations:** 1 Buea Regional Hospital, Buea, Cameroon; 2 Clinical Research Education, Networking and Consultancy, Douala, Cameroon; 3 Athena Institute for Research on Innovation and Communication in Health and Life Sciences, Vrije Universiteit Amsterdam, Amsterdam, The Netherlands

**Keywords:** Ventricular tachycardia, Case report, Defibrillation, Sub-Saharan Africa

## Abstract

**Background:**

Ventricular tachycardia is a life threatening cardiac arrhythmia. It needs management with defibrillation, without which, immediate death may occur.

**Case presentation:**

A 66 year old black African patient with a 2 year history of hypertension was admitted to the emergency department of the Buea Regional hospital, a semi-urban setting in Cameroon, after presenting with syncope while in church. The wife described a similar episode 2 weeks prior without any further evaluation. Upon arrival at the emergency, patient had regained consciousness but lethargic, tachypneic and diaphoretic. The blood pressure was 85/61 mmHg; the pulse was 219/min, weak and thready. He had cold extremities. A 12 lead electrocardiogram performed showed a sustained monomorphic ventricular tachycardia at 230/min. He was administered six tablets of amiodarone, oxygen by nasal cannula and intravenous fluids. No electrical cardioversion was attempted due to the non availability of a defibrillator. Outcome was fatal with death of the patient 30 min after his arrival to the emergency.

**Conclusion:**

Our health facilities should be well equipped for resuscitative measures by adopting Advanced Cardiac Life Support as cardiovascular diseases are becoming more frequent in our settings.

## Background

Ventricular tachycardia (VT) is a potentially life-threatening arrhythmia which most commonly occurs in patients with structural heart disease. Ventricular tachycardia can be classified into monomorphic and polymorphic rhythms. When the QRS complexes remain identical from beat to beat it is classified as monomorphic, suggesting a stable origin of tachycardia from a focus or a structural substrate [1]. This case describes a man who presented with hemodynamic collapse and syncope induced by a sustained monomorphic ventricular tachycardia in a semi-urban setting in Cameroon. Limitations in management options with lack of advanced cardiac life support led to the death of the patient.

## Patient and case report

**Fig. 1:**
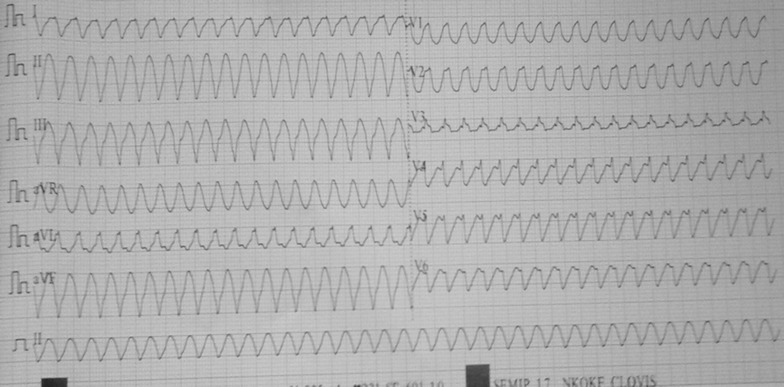
12 lead electrocardiogram showing a sustained monomorphic ventricular tachycardia at 230/min

A 66 year old black African male patient was admitted to the emergency department of the Buea Regional Hospital in Cameroon, after presenting with syncope while he was in church, standing for a worship service. He had a past history of hypertension of 5 years and smoking which he stopped since 10 years. There were no other known risk factors for heart disease. His anti-hypertensive medications consisted of atenolol and hydrochlorothiazide. His wife described a similar episode of syncope 2 weeks prior for which he consulted his primary care provider without further evaluation for the syncope. He was brought to the hospital about 15 min later without any initial resuscitative attempts at the church premises. Upon arrival at the emergency, patient had regained consciousness but lethargic, tachypneic and diaphoretic. The blood pressure was 85/61 mmHg; the pulse was 219/min, weak and thready. He had cold extremities. The heart sounds were very faint and rapid. The lung examination was normal A 12 lead electrocardiogram performed showed a monomorphic ventricular tachycardia at 230/min (Fig. 1). He was administered six tablets of amiodarone, the only anti-arrhythmic available in an attempt to cardiovert, oxygen by nasal cannula and intravenous fluids. No electrical cardioversion was attempted due to absence of a defibrillator. Outcome was fatal with death of the patient 30 min after his arrival to the emergency department.

## Discussion

We have reported a case of fatal sustained monomorphic ventricular tachycardia in a 66-year old male patient with a history of hypertension in semi-urban setting in Cameroon.

Ventricular tachycardia is a life threatening ventricular arrhythmia. The most common setting of ventricular tachycardia is ischemic heart disease in which myocardial scar tissue is the substrate for reentry activity [2]. The most common cause of scar is an old infarct. We did not have a previous electrocardiogram which could give clues to the underlying cause of ventricular tachycardia in this patient. Our patient was older, and had hypertension which increases the likelihood of ischemic heart disease although ventricular tachycardia can also occur in the absence of structural heart disease. Although ischemic heart disease was considered to be rare in sub-Saharan African, recent evidence suggests ischemic heart disease is by no means rare in Africans [3, 4]. An increase in the number of trained cardiologists and diagnostic tools (electrocardiogram, cardiac ultrasound), though sub-optimal, could partly explain this reported increase in the incidence of ischemic heart disease. Although heart diseases are on the rise in sub-Saharan Africa, the diagnostic and therapeutic facilities are largely inadequate thus making emergency treatment difficult as it was in the case presented.

Despite being the leading cause of death worldwide, guidelines and evidence supporting management of cardiovascular diseases have limitations in resource limited settings. Developing countries face a rapidly increasing and disproportionate burden of cardiovascular disease yet differences in setting and resource limitations bring challenges that have a major influence in management options—especially with routine investigations for diagnosis and interventional considerations. In addition, general awareness, diagnosis and management of some cardiovascular emergencies remain poor.

In patients with ventricular tachycardia, mortality correlates with the degree of structural heart disease. Underlying structural heart diseases have been associated with the degeneration of ventricular tachycardia to ventricular fibrillation. Regardless of the etiology, emergent cardioversion is warranted for sustained ventricular tachycardia that is causing symptomatic hypotension, pulmonary edema, or myocardial ischemia [5]. Providing effective emergency and urgent care is a considerable challenge in resource limited countries. Difficulties exist with regard to transportation, communications, equipment, facility infrastructure, medication supply lines, affordability and availability of skilled healthcare providers. Historically, infectious diseases have been the major contributors to morbidity and mortality in resource-limited settings. However non communicable diseases including cardiovascular diseases are rapidly increasing and have become major contributors to morbidity and mortality in developing countries.

Sustained ventricular tachycardia may lead to hemodynamic collapse. Consequently, these patients require urgent conversion to sinus rhythm. The strategy for conversion depends on whether the patient is hemodynamically stable or unstable.

Ventricular tachycardia at rates below 150 beats/min is frequently well tolerated, whereas VT at rates above 200 beats/min are usually unstable. Acutely unstable patients—as evidenced by altered consciousness, severe hypotension, or other signs of significant end-organ hypoperfusion—require prompt electrical cardioversion. Stable patients have adequate vital end-organ perfusion and thus do not experience signs or symptoms of hemodynamic compromise. Treatment depends on whether the VT is monomorphic or polymorphic and whether left ventricular function is normal or impaired. In stable patients with monomorphic VT and normal left ventricular function, restoration of sinus rhythm is typically achieved with intravenous procainamide or sotalol. Lidocaine may also be used. If left ventricular function is impaired, amiodarone (or lidocaine) is preferred to procainamide for pharmacologic conversion because of the latter drug’s potential for exacerbating heart failure. However, current evidence indicates that amiodarone should not be the first-line antiarrhythmic for stable VT, because its effects on myocardial conduction and refractoriness are gradual in onset [6, 7]. In our case report, there was no defibrillator available in the hospital as a result; cardioversion was attempted with oral amiodarone associated with supportive resuscitative measures with oxygen. This however proved ineffective with the death of the patient.

## Conclusion

Cardiac diseases and ventricular tachycardia are increasing in developing countries. Our health facilities should be well equipped for resuscitative measures by adopting advanced cardiac life support.

## Abbreviation

VT: ventricular tachycardia
